# LRRK2 Mutations and Asian Disease-Associated Variants in the First Parkinson's Disease Cohort from Kazakhstan

**DOI:** 10.1155/2020/2763838

**Published:** 2020-02-19

**Authors:** Rauan Kaiyrzhanov, Akbota Aitkulova, Chingiz Shashkin, Nazira Zharkinbekova, Mie Rizig, Elena Zholdybayeva, Zharkyn Jarmukhanov, Vadim Akhmetzhanov, Gulnaz Kaishibayeva, Talgat Khaibullin, Altynay Karimova, Serik Akshulakov, Askhat Bralov, Nurlan Kissamedenov, Zhanar Seidinova, Anjela Taskinbayeva, Aliya Muratbaikyzy, Henry Houlden

**Affiliations:** ^1^University College London, Institute of Neurology, Department of Neuromuscular Disorders, Queen Square, WC1N 3BG, London, UK; ^2^National Center for Biotechnology, Department of Molecular Genetics, 13/5 Korgalzhyn Avenue, 01000 Nur-Sultan, Kazakhstan; ^3^South Kazakhstan Medical Academy, Department of Neurology, 1/1Al-Farabi Avenue, 160019 Shymkent, Kazakhstan; ^4^Institute of Neurology Named After S. K. Kaishibayev, 9a Mamur 4 Micro-district, 050000 Almaty, Kazakhstan; ^5^Semey Medical University, Department of Neurology, 103 Abai Street, 071400 Semey, Kazakhstan; ^6^National Center for Neurosurgery, 34/1 Turan Avenue, 01000 Nur-Sultan, Kazakhstan

## Abstract

**Background:**

LRRK2 mutations have emerged as the most prevalent and potentially treatable determinants of Parkinson's disease (PD). Peculiar geographic distribution of these mutations has triggered an interest in genotyping PD cohorts of different ethnic backgrounds for LRRK.

**Objective:**

Here, we report on the results of LRRK2 screening in the first Central Asian PD cohort.

**Methods:**

246 PD patients were consecutively recruited by movement disorder specialists from four medical centers in Kazakhstan, and clinicodemographic data and genomic DNA from blood were systematically obtained and shipped to the Institute of Neurology University College London together with DNAs from 200 healthy controls. The cohort was genotyped for five LRRK2 mutations (p.Gly2019Ser, p.Arg1441His, p.Tyr1699Cys, p.Ile2020Thr, and p.Asn1437His) and three East Asian disease-associated variants (p.Gly2385Arg, p.Ala419Val, and p.Arg1628Pro) via Kompetitive allele-specific polymerase chain reaction assay analysis.

**Results:**

None of the study subjects carried LRRK2 mutations, whereas the following Asian variants were found with insignificant odds ratios (OR): p.Gly2385Arg (1.2%, minor allele frequency (MAF) 0.007, OR 1.25, *p*=0.8), p.Ala419Val (3.7%, MAF 0.02, OR 1.5, *p*=0.8), p.Ala419Val (3.7%, MAF 0.02, OR 1.5,

**Conclusions:**

We showed that East Asian LRRK variants could be found in Central Asian populations but their pathogenicity remains to be elucidated in larger PD cohorts.

## 1. Introduction

The important recent achievement in Parkinson's disease (PD) research has been the identification of several causative and risk genes with their putative functions. Leucine-rich repeat kinase 2 (LRRK2) gene has been distinguishable from other known PD genes with a number of functional and epidemiologic features. LRRK2 is a highly conserved and widely expressed gene that encodes a unique multifunctional and multidomain protein, named dardarin [1]. Dardarin is a complex chain of 2 527 polypeptides containing two distinct enzymes, namely protein kinase and guanosine triphosphatase (GTPase), as well as multiple protein interaction domains [2]. These domains might interact with each other and other cell signaling proteins, thus playing a putatively key role in cellular function [3–5].

Intriguing is the fact that almost every LRRK2 domain is susceptible to PD-associated mutations resulting largely in idiopathic PD- (iPD-) like phenotype and pleomorphic neuronal pathology [2, 4]. Amongst the number of known LRRK2 mutations, p.Gly2019Ser mutation has emerged as an important determinant of familial autosomal dominant PD and iPD in North African Arabs, Ashkenazi Jews, and to a lesser degree in European and North American populations [6].

Interestingly, it appears that some LRRK2 mutations and disease-associated variants are specific to particular ethnic groups, most likely due to common founder effects [7]. This is evidently applicable to p.Gly2019Ser mutation, which is common in PD patients from the Western hemisphere and has not yet been reported in the big PD cohorts from East Asia. Similarly, several PD risk variants of LRRK2 including p.Gly2385Arg, p.Ala419Val, and p.Arg1628Pro have only been reported in East Asian populations and have been absent in the Western PD cohorts [8].

PD genetics has been largely unexplored in several world regions, including Central Asia. Here, we investigate 8 LRRK2 mutations and East Asian risk variants in an interesting population residing between Europe and Asia, in the first cohort of PD patients and healthy controls from Kazakhstan.

## 2. Study Methodology

### 2.1. Study Subjects

A total of 246 PD patients were consecutively recruited, with no regard to nationality, during 14 months from the National Center for Neurosurgery in Nur-Sultan city, movement disorders clinics in Almaty city, and a regional hospital in Shymkent city in Kazakhstan. The diagnoses of clinically established and clinically probable PD were made on the basis of the agreement between two movement disorder specialists according to the Movement Disorders Society (MDS) PD criteria [9]. Both iPD and familial PD cases with their available first-degree relatives were included in the study. Clinicodemographic characteristics of the cohort were uploaded to University College London (UCL) Research Data Capturing Database (Redcap) online secure database and its summary is given in Table 1. There were 21 patients (8.5%, 21/246) with a family history of PD and/or tremor, and 31 (12.6%, 31/246) patients with the onset of PD before 40 years of age. The male to female ratio was 0.95 : 1. The mean age of PD onset was 55.06 ± 11.15 (range 14–77), and the mean age at the last examination was 61.7 ± 10.3 (range 28–83). Self-reported nationalities in 72.8% of the cohort were Kazakh, 20.7% were Russian, and the remaining nationalities were Uyghur (2.8%), Tatar (2%), Korean (1.3%), and Tajik (0.4%). Genomic DNAs of age- and gender-matched 200 unrelated control subjects were obtained from the research-ready database of neurologically healthy Kazakhs from the National Center for Biotechnology, Nur-Sultan (NCB).

This study was approved by the Research Ethics Committee of NCB (4/29.08.2017) and the Institute of Neurology University College London (IoN UCL) (07/Q0512/26). Written informed consent for participation in the study was obtained from each subject. All personal information was hidden with unique study identifiers.

### 2.2. Genetic Analysis

Whole venous blood was collected from the subjects at the recruitment centers and sent to NCB for DNA extraction using the standardized laboratory protocols. DNAs were then shipped to IoN UCL for genetic analysis. At IoN, DNAs were checked for quality and concentrations using NanoDrop Spectrophotometer (Thermo Scientific, Waltham, MA, USA). Samples were uniformly diluted to 25 ng/*μ*l, and 25 *μ*l volume of DNA from each sample was transferred to 96-well plates.

**Figure 1:**
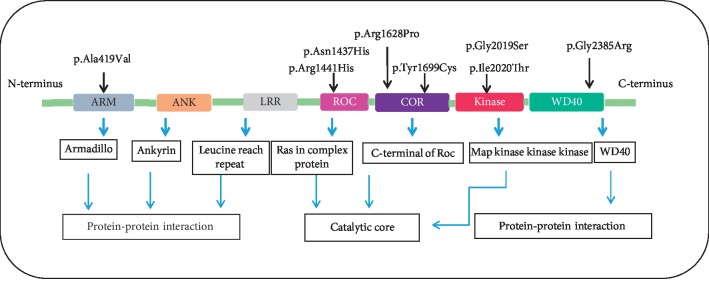
LRRK2 protein: functional domain and the localization of 8 variants. ANK-ankyrin repeat; ARM-armadillo; LRR-leucine-rich repeat; ROC-Ras of complex proteins: GTPase; COR-C-terminal of ROC; WD40-WD-40 domain. Pathogenic mutations are highlighted in blue, and East Asian disease-associated variants are highlighted in red.

On the basis of the literature review, 8 LRRK2 single-nucleotide polymorphisms (SNPs) of interest were selected for the analysis. Five of them are commonly reported LRRK2 mutations: (c.6055G>A) p.Gly2019Ser (rs34637584), (c.4322G>A) p.Arg1441His (rs34995376), (c.5096A>G) p.Tyr1699Cys (rs35801418), (c.6059T>C) p.Ile2020Thr (rs35870237), and (c.4309A>C) p.Asn1437His (rs74163686); and three of them are the East Asian-specific PD-associated variants: (c.7153G>A) p.Gly2385Arg (rs34778348), (c.1256C>T) p.Ala419Val (rs34594498), and (c.4883G>C) p.Arg1628Pro (rs33949390) (Figure 1). The SNPs and surrounding 50 base-pairs were annotated in Ensembl genome browser (see S1 in the supplementary material for comprehensive analysis). The design of primers, adaptation of assays, and genetic analysis were performed in LGC Genomics, England. No positive and negative controls were available for the selected LRRK2 SNPs. LRRK2 genotyping was done using Kompetitive Allele-Specific Polymerase chain reaction (PCR) genotyping assay (KASP™, LGC Genomics. Herts, UK), a method that enables biallelic scoring of SNPs and insertion and deletions at specific loci through competitive allele-specific PCR. Genotyping followed the LGC Genomics protocol. SNP viewer Software (version 1.99, Hoddesdon, UK) was used to visualize the genotyping results (https://www.biosearchtech.com/support/tools/genotyping-software/snpviewer). Familial and young-onset PD cases with positive LRRK2 substitutions were sent for exome sequencing (WES) to Macrogen, South Korea. In addition, when LRRK2 mutations were identified in a proband, Sanger sequencing was performed for all available family members. Thus, we enrolled 13 additional living relatives for the p.Gly2385Arg and p.Ala419Val mutations from two families.

### 2.3. Statistical Analysis

Statistical analysis was performed using IBM SPSPS version 21 (Chicago, USA). Genotype frequency distributions were tested for conformity to Hardy–Weinberg equilibrium (HWE). *T*-test and chi-squared tests were used at the level of significance 0.05. Odds ratios (OR) were calculated and presented with 95% confidence interval (CI) values.

## 3. Results

### 3.1. KASP Coverage

1.6–3.2% of samples were uncalled in KASP assay analysis and this was within the expected values [10]. The number of uncalled samples for each LRRK2 SNP is shown in Supplementary Materials ([Supplementary-material supplementary-material-1] and [Supplementary-material supplementary-material-1] for comprehensive analysis).

### 3.2. SPNs with Negative Findings

The following most pathogenic LRRK2 mutations p.Gly2019Ser, p.Arg1441His, p.Tyr1699Cys, p.Ile2020Thr, and p.Asn1437His were not found in our cohort of PD patients and controls. All of the 246 PD subjects and 200 controls were homozygous for wild-type alleles of these SNPs (Table 2). The allelic frequencies for the aforementioned SNPs were in Hardy–Weinberg equilibrium (*p*=1).

### 3.3. SNPs with Positive Findings

#### 3.3.1. p.Gly2385Arg (c.7153G>A)

**Figure 2:**
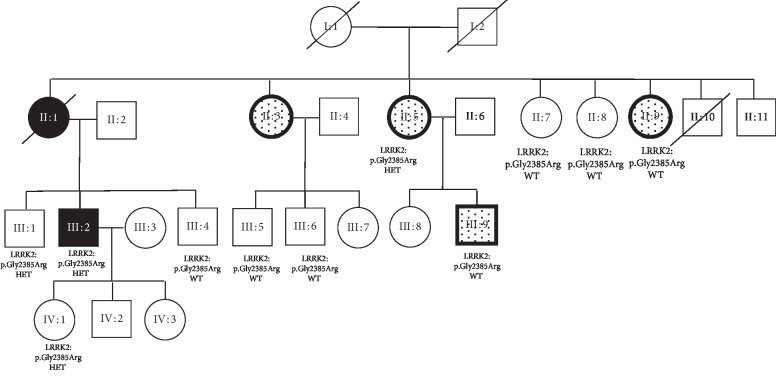
Familial case with p.Gly2385Arg variant. III:2 Proband 40 years old, PD onset at 38 years. II:1 Affected mother of the proband. PD onset at 55 years. Died at 58 years. Levodopa responsive PD. II:3 affected maternal aunt. 10 years' history of unilateral right-hand tremor at rest and action. II:5 Affected maternal aunt, 54 years old. 10 years' history of positional unilateral tremor. III:9 son of II:5. Right-hand positional tremor. II:9 Affected maternal aunt, 4-5 years' history of right-hand positional tremor.

Monoallelic p.Gly2385Arg variant (rs34778348) was found in three PD cases (1.2%, *n* = 3/239) and two controls (1%, *n* = 2/199) (Table 3). The first positive case was a young Kazakh male with an autosomal dominant family history of PD. He developed PD at the age of 38 years, and the onset symptoms were depression, anxiety, hyposmia, and left-hand tremor. He had an affected mother, who had developed PD at the age of 55 years and deceased at the age of 58 years. Interestingly, his mother had three female siblings with upper-limb tremor but no signs of bradykinesia (Figure 2). He had fast progression in the disease course and in two years from the disease onset developed difficulty in rising up from a chair, freezing episodes, urinary frequency, and gait abnormalities. His off-stage MDS UPDRS motor scale score was 79 with Hoehn–Yahr stage 3. There was a good response to levodopa. WES in the proband did not reveal any other known PD genes. p.Gly2385Arg variant was tested in six of his healthy relatives and in those with tremor (*n* = 4) by Sanger sequencing. The variant was present in one of the two unaffected siblings of the proband, unaffected 20-year-old daughter of the proband, and in only one out of the four relatives with tremor (Figure 2).

The second PD case was a 71-year-old Kazakh female with sporadic PD onset at the age of 66 years. She had a mild and slowly progressive disease course. Her off-stage MDS UPDRS motor score was 16 and Hoehn–Yahr stage 1. Dopamine agonists effectively controlled her motor symptoms.

The third PD case was a 65-year-old Kazakh female with sporadic PD with the onset at the age of 61 years. She had also a mild disease course and was not on levodopa. Her off-stage MDS UPDRS motor score was 14 and Hoehn–Yahr stage 2.

p.Gly2385Arg positive healthy controls were 50-year-old and 54-year-old male and female subjects. The allelic and genotypic frequencies for p.Gly2385Arg were in Hardy–Weinberg equilibrium (*p*=0.9) and did not statistically differ between cases and controls (OR 1.25, 95% C.I.: 0.2071–7.5688, *p*=0.8) (Tables 2 and 4).

#### 3.3.2. p.Ala419Val (c.1256C>T)

**Figure 3:**
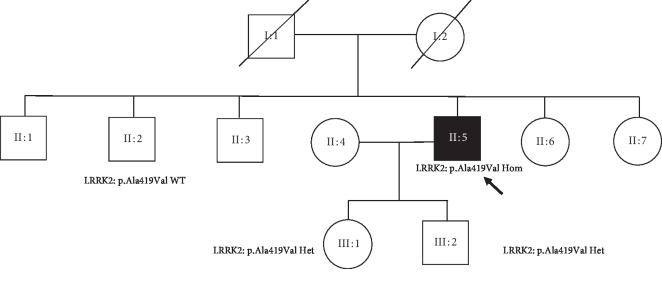
Homozygous p.Ala419Val proband and his family tree.

LRRK2 p.Ala419Val variant (rs34594498) was positive in 9 PD cases (3.7%, *n* = 9/242) and 5 controls (2.5%, *n* = 5/199), giving the OR of 1.5 (95% C.I.: 0.4941–4.5463, *p*=0.4) (Table 4). WES in the probands with young-onset and familial PD did not reveal any other known PD genes. One positive case had homozygous substitution in c.1256C>T (T/T). This was a 52-year-old Kazakh patient with the onset of sporadic PD at the age of 48 years. The patient expressed akinetic-rigid PD with a good response to levodopa but early and severe motor complications. He reached HY stage 3 in four years from the onset of motor symptoms. Two unaffected children of the proband were heterozygous for LRRK2 c.1256C>T substitution, whereas one unaffected sibling of the proband did not have LRRK2 c.1256C>T substitution on Sanger sequencing segregation analysis (Figure 3) (see [Supplementary-material supplementary-material-1] in the supplementary material for comprehensive image analysis).

Five out of the 9 LRRK2 p.Ala419Val carriers developed PD before the age of 50 years, the youngest manifestation being at the age of 26 years. The mean age at onset for the p.Ala419Val carriers was 48.3 ± 12.6 (range 26–69) and this did not significantly differ from the noncarriers (48.3 ± 12.6 v54.7 ± 12.3, *p*=0.19). The mean age at examination was 57.4 ± 12.9 (range 32–82), and mean disease duration was 9.1 ± 6.1 (range 2–20) (Table 5). The mean HYS score for the positive cases was 2.5 ± 0.5.

Interestingly, self-reported nationalities in three out of the nine positive cases were Russian, one case was half Kazakh and half Russian, and the remaining 5 cases were Kazakhs. Two of the Russian patients had an autosomal dominant history of PD with onset after the age of 50 years. All of the LRRK2 p.Ala419Val-positive cases had a good response to levodopa. The allele frequencies for LRRK2 p.Ala419Val in PD cases deviated from Hardy–Weinberg equilibrium (*p*=0.004) (Table 2).

#### 3.3.3. p.Arg1628Pro (c.4883G>C)

PD cases were negative for p.Arg1628Pro (rs33949390) variant, whereas two control subjects were found to be positive (1%, *n* = 2/199). The variant was in Hardy–Weinberg equilibrium (*p*=0.9) in controls.

## 4. Discussion

This study screened cohorts of PD patients and controls from Kazakhstan for five LRRK2 mutations and three Asian disease-associated variants. To date, over 80 LRRK2 disease-causing and disease-associated variants have been described in literature since the gene was discovered in 2004. However, only eight of them have been acknowledged as PD-causing mutations including p.Gly2019Ser, p.Arg1441His, p. Arg1441Cys, p.Arg1441Gly, p.Tyr1699Cys, p.Ile2020Thr, p. Asn1437His, and p. Ile2012Thr. All of these mutations affect the catalytic core of the LRRK2 enzyme [11]. Among these mutations, p.Gly2019Ser is the most common followed by the substitution of arginine (Arg) by glycine (Gly), cysteine (Cys), or histidine (His) in the position 1441 of LRRK2 gene [11]. p.Gly2019Ser mutation has a global prevalence of 1% in patients with iPD and around 4% in familial PD. It is noteworthy that p.Gly2019Ser has been predominantly reported in the North African population where it is responsible for 30–42% of familial and 30–34% of sporadic PD cases. Its prevalence shows high figures in Ashkenazi Jews (28% of familial PD and 10% in iPD) and among European as well as North American populations (6% and 3%, respectively) [12]. Conversely, p.Gly2019Ser mutation has not been reported in Asians (<0.1%) [13, 14]. p.Gly2019Ser was reported in Russian PD cohorts (%), possibly in subjects of Ashkenazi Jewish origin [15].

The currently known most deleterious LRRK2 mutations associated with PD were not found in our cohort. The absence of these mutations in our cohort is similar to other Asian studies [6] This is probably due to the founder effects of these mutations, which seem to be specific to Western populations.

### 4.1. p.Gly2385Arg

LRRK2 p.Gly2385Arg substitution is located in the WD-40 domain, a toroidal beta-propeller structure responsible for protein-protein interactions [16]. There have been 5 independent case-control studies reporting this variant [6, 13, 16–18] and 14 studies screening their cohorts for p.Gly2385Arg [14, 19].

Originally, p.Gly2385Arg variant was identified in a small Taiwanese PD family, in a proband and his affected father [20]. Later, a number of studies in Chinese, Taiwanese, Korean, and Japanese populations have reported p.Gly2385Arg variant significantly more among PD patients than controls, with minor allele frequency (MAF) up to 0.4 in patients (Table 6). Thus, p.Gly2385Arg variant was attributed to a risk factor for sporadic and familial PD. The population attributable risk for p.Gly2385Arg in Han-Chinese ethnicity was estimated to be around 4% [18], and the variant probably originated around 4800 years ago from one ancestor [16]. p.Gly2385Arg variant had a tendency for equal distribution across genders and age groups [6]. Regarding the clinical presentation, p.Gly2385Arg carriers expressed typical PD and homozygous cases were not clinically different from heterozygotes and non-carriers [13]. This variant seems not to influence the age of PD onset, as the mean ages of onset in p.Gly2385Arg carriers and non-carriers have not been consistently reported to significantly differ between these groups [6, 13, 17]. Reports on the association of p.Gly2385Arg carrier status with a family history of PD have also been inconsistent [16, 17].

In regards to non-Chinese populations, p.Gly2385Arg was found in 1.2% (2/166) of PD patients and 0.6% (2/306) of controls in Malay/Indian ethnicity from Singapore [8] The frequency of p.Gly2385Arg in cases and controls was not significantly different in this Singaporean study, and considerably lower than the frequency of 8–10% reported in Chinese PD subjects [6, 8]. A recent study on Malaysian PD subjects has found a significant association between p.Gly2385Arg and increased risk of PD [14] The variant was absent in 405 Iranian subjects [22] and positive in only one individual of Northern European origin among 14,002 screened Caucasian subjects [25].

Evidence from functional studies suggests that p.Gly2385Arg substitution leads to the replacement of hydrophobic glycine with the hydrophilic arginine and increases the net positive charge on the 40WD domain of LRRK2. Both LRRK2 proteins with wild-type and p.Gly2385Arg variant localized to the cytoplasm forming aggregates, but the intensity of apoptosis is higher in p.Gly2385Arg variant under oxidative stress conditions [18].

The frequency of p.Gly2385Arg variant in our study (1.2% patients and 1% controls) was almost similar to non-Chinese Singaporean subjects. If MAF for Gly2385Arg in Chinese and Japanese PD populations was between 0.05 and 0.4 (Table 6), MAF in our study was 0.007. This might suggest that p.Gly2385Arg could be found in non-Chinese Asians but in considerably less proportion. Due to a small amount of non-Chinese subjects screened for p.Gly2385Arg and its equal distribution between patients and healthy controls, currently, it is difficult to ascertain the role of this LRRK2 variant in the risk of PD among Central Asian populations. Contemporary data suggest that p.Gly2385Arg could be a risk factor for PD only in selected Asian races.

On the other hand, among three patients positive to p.Gly2385Arg in our study, we had an interesting familial PD case, where proband and his deceased mother had PD, whereas maternal siblings and one maternal cousin of the proband had unilateral asymmetric UL tremor. Although all other known PD genes have been excluded by WES in the proband, p.Gly2385Arg did not completely segregate in the family, being positive in some unaffected family members and negative in some affected (Figure 2). To date, the variant has been shown to segregate with PD in only one small Taiwanese family with affected proband, affected father of the proband, and unaffected sibling. We showed the segregation of p.Gly2385Arg, although incomplete, in a larger family presenting not only with PD but tremor. The incomplete segregation in our family could be due to reduced penetrance or other unknown genetic factors.

### 4.2. p.Ala419Val

There have been nine reports describing p.Ala419Val variant [26]. The variant resides in the LRRK2 Armadillo domain and is predicted deleterious by online prediction tools with high conservation in the vertebrates [26].

Initially, the association between this variant and PD was described by Ross et al. [23] where p.Ala419Val was tested in 2,338 Asian subjects from Japan, Korea, and Taiwan (1,376 PD cases and 962) in a large-scale multicenter study. The study results revealed the OR of 2.27 (95% CI: 1.35–3.83, *p*=0.0011). Several studies before and after the reported association of p.Ala419Val with PD have found either no carriers of this variant in large cohorts of PD patients and controls or insignificant OR (Table 7), thus considering the variant as putatively nonpathogenic population-specific SNP.

The MAF for p.Ala419Val in PD patients has been 0.002–0.018 in Chinese, and 0.026–0.029 in Japanese and Korean populations in studies reporting positive association [23, 31]. Interestingly, the replication studies in the Chinese and Taiwanese ethnicities have yielded inconsistent results. While p.Ala419Val was negative or positive with insignificant OR in some studies, several studies and their meta-analysis reported a significant association between this variant and predominantly early-onset PD (Table 7, [26]). This has been explained by possible natural sampling variation and population heterogeneity [30] on the one hand. On the other hand, Li et al. [26] argued that the discrepancy is likely to result from different mean ages at onset (AAO) of PD patients in these studies. Thus, while the mean AAO in p.Ala419Val-positive reports on Chinese ethnicity was <55 years [23, 26, 29], negative reports on the same population had AAO above 60 years [28, 30, 31, 33]. Provided the fact that p.Ala419Val might have a strong association with early-onset Chinese PD, the likelihood of yielding positive findings could be higher in young-onset PD cohorts.

The MAF for p.Ala419Val in PD patients in our study was 0.02, which is higher than in Chinese and Taiwanese populations and closer to Japanese and Korean (Table 7). Taking into account the young mean AAO in our Kazakhstani PD cohort (55.06 ± 11.15) and reference to Li et al. [26], one might explain the high MAF for p.Ala419Val. Moreover, the AAO in p.Ala419Val-positive PD patients was remarkably young (48.3 ± 12.6) in our study. However, the high frequency (MAF 0.012) of p.Ala419Val in healthy Kazakhstani controls, who also had a mean age of below 55 years, and insignificant OR do not allow us to ascertain the pathogenicity of p.Ala419Val in Kazakhstani PD.

We have found a previously unreported homozygous carrier of p.Ala419Val variant with PD onset before 50 years and relatively aggressive disease course. If this LRRK2 variant is nonpathogenic and not rare in our population, the likelihood of p.Ala419Val homozygous carriers would increase and this might result in the HWE deviation.

p.Ala419Val variant seems to be present not only in Kazakhs but also in self-reported Russian patients with late-onset or familial PD. Considering the reported specificity of p.Ala419Val to Asian populations, we could speculate that these self-reported Russian subjects in our study could have a mixed ethnic background, particularly with Tatars, an Asian population with some Russian phenotypic features. Alternatively, the variant could also be present in the Russian population.

### 4.3. p.Arg1628Pro

Regarding p.Arg1628Pro, data from a meta-analysis, including 19 studies with a total of 9,927 PD patients and 8,602 controls, suggest that the variant is significantly associated with the risk of PD in East Asian populations [34]. We failed to find this variant in our PD patients but it was present in controls. This might suggest that p.Arg1628Pro could be a common benign polymorphism in Kazakhstani population.

We have to acknowledge the limitations in our study due to the relatively small sample size, the nonhomogeneous ethnic composition of the PD cohort, as only 72.8% of the PD cohort were Kazakhs. In addition, controls were not perfectly matched to cases by age, gender, and ethnicity.

## 5. Conclusions

The negative findings on common LRRK2 PD causing mutations, and the presence of LRRK2 Asian-specific variants in our PD patients, although at insignificant level compared with controls, suggest that further large-cohort genetic studies are required in Central Asia to ascertain the pathogenicity of LRRK2 Asian-specific variants in the Central Asian PD population.

## Figures and Tables

**Table 1:** Clinical and demographic characteristics of the cohort.

	Cases	Controls
Number	246	200

Ethnic groups, abs number (%)	Kazakhs 179 (72.8%)	
	Russians 51 (20.7%)	
	Uygurs 7 (2.8%)	
	Tatars 5 (2%)	
	Koreans 3 (1.3%)	
	Tajiks 1 (0.4%)	

Sex distribution	Males—120, females—126 M : F ratio−0.95 : 1	Males—62, females—138 M : F ratio 0.4 : 1

Age at examination (mean)	**61.7** ± 10.3 (range 28–83) For males—60.3 ± 10.6 (range 28–82), *p*=0.03 For females—63.1 ± 9.9 (range—32–83)	Mean age—**54.93** ± 4.8 (47–66) For males—**55.27** ± 4.8 (47–66) For females—**54.78** ± 4.7 (47–65)

Age of onset (mean)	**55.06** ± 11.15 (range 14–77) For males—53.3 ± 11.9 (range 14–76), *p*=0.01 For females—56.8 ± 9.9 (range 26–77)	

Disease duration (mean)	**13.2** ± 9.3 (range 1–24)	

HY stage off (mean)	**2.4** **±** 0.6 (range 1–5) For males—2.3 ± 0.7 *p*=0.6 For females—2.4 ± 0.6	

Family history of PD and tremor, abs number (%)	**21 (8.5%)**	

Young-onset cases, abs number (%)	**31 (12.6%)** before the age of 40 **65 (26.4%)** before the age of 50	

M : F–male to female.

**Table 2:** Allele frequency and distribution of the 8 tested LRRK2 variants.

SNP	Hardy–Weinberg equilibrium *p*-value	Number of samples	Allele	*n* ^a^	Frequency	Genotype	*n* ^b^	Frequency
p.Gly2019Ser (c.6055G>A)	1	198 controls				GG	198	1.00
			G	396	1.00	GA	0	0.00
			A	0	0.00	AA	0	0.00
	1	241 PD		482	1.00	GG	241	1.00
			G	0	0.00	GA	0	0.00
			A			AA	0	0.00

p.Arg1441His (c.4322G>A)	1	199 controls				GG	199	1.00
			G	398	1.00	GA	0	0.00
			A	0	0.00	AA	0	0.00
	1	240 PD		480	1.00	GG	240	1.00
			G	0	0.00	GA	0	0.00
			A			AA	0	0.00

p.Tyr1699Cys (c.5096A>G)	1	196 controls				GG	196	1.00
			G	392	1.00	GA	0	0.00
			A	0	0.00	AA	0	0.00
	1	239 PD		478	1.00	GG	239	1.00
			G	0	0.00	GA	0	0.00
			A			AA	0	0.00

p.Ile2020Thr (c.6059T>C)	1	198 controls				TT	198	1.00
			T	396	1.00	TC	0	0.00
			C	0	0.00	CC	0	0.00
	1	242 PD		484	1.00	TT	242	1.00
			T	0	0.00	TC	0	0.00
			C			CC	0	0.00

p.Asn1437His (c.4309A>C)	1	199 controls				AA	198	1.00
			A	398	1.00	AC	0	0.00
			C	0	0.00	CC	0	0.00
	1	240 PD		480	1.00	AA	240	1.00
			A	0	0.00	AC	0	0.00
			C			CC	0	0.00

p.Gly2385Arg (c.7153G>A)	0.94	199 controls				GG	197	0.99
			G	396	0.995	GA	2	0.01
			A	2	0.005	AA	0	0.00
	0.92	239 PD				GG	236	0.99
			G	475	0.993	GA	3	0.01
			A	3	0.007	AA	0	0.00

p.Ala419Val (c.1256C>T)	0.85	199 controls				CC	194	0.98
			C	393	0.988	CT	5	0.02
			T	5	0.012	TT	0	0.00
	0.004	242 PD			0.98	CC	233	0.97
			C	474	0.02	CT	8	0.04
			T	10		TT	1	0.00

p.Arg1628Pro (c.4883G>C)	0.94	199 controls				GG	197	0.99
			G	396	0.995	GT	2	0.01
			C	2	0.005	TT	0	0.00
	1	236 PD				GG	236	1.00
			G	472	1.00	GT	0	0.00
			C	0	0.00	TT	0	0.00

*n*1—number of alleles, *n*2—number of genotypes, PD–Parkinson's disease, HWE–Hardy–Weinberg equilibrium, and NA–not applicable.

**Table 3:** p.Gly2385Arg-positive PD patients and their characteristics.

	Number (246–7 uncalled = 239)	Mean age at examination	Mean age of onset	Mean disease duration	Family history	Mean HY stage off
Carriers	3	58.6 ± 13.4	55 ± 12.1	4 ± 0.8	1	2 ± 0.8
Noncarriers	236	61.7 ± 10.3	55 ± 11.1	6.8 ± 4.7	20	2.3 ± 1.5
*p* value		**0.77**	**0.99**	**0.01**		**0.56**

**Table 4:** The allelic frequency and odds ratios for the positive LRRK2 Asian disease-associated variants.

SNP	Nucleotide change	Amino acid change	MAF		Or (95% CI)	*p* value
			PD (*n* = 239^a^, 242^b^, 236^c^)	Controls (*n* = 199)		
rs34778348	c.7153G>A	p.Gly2385Arg^a^	0.007 (A)	0.005 (A)	1.25 (0.2071–7.5688)	0.8

rs34594498	c.1256C>T	p.Ala419Val^b^	0.02 (T)	0.012 (T)	1.5 (0.4941 – 4.5463)	0.4
rs33949390	c.4883G>C	p.Arg1628Pro^c^	0.0 (C)	0.005 (C)	NA	NA

MAF–minor allele frequency, NA–not applicable, OR–odds ratio, and PD–Parkinson's disease.

**Table 5:** p.Ala419Val-positive PD patients and their characteristics.

	Number 246-4uncalled = 242	Mean age at examination	Mean age of onset	Mean disease duration	Family history	Mean HY stage off
Carriers	9	57.4 ± 12.9	48.3 ± 12.6	9.1 ± 6.1	2	2.5 ± 0.5
Noncarriers	233	61.6 ± 10.4	54.7 ± 12.3	6.8 ± 7	19	2.2 ± 0.7
*P* value		**0.38**	**0.19**	**0.3**		**0.02**

HY–Hoehn–Yahr.

**Table 6:** Studies investigating LRRK2 p.Gly2385Arg variant in Asian populations.

	Cases	Controls	Ethnicity	OR	MAF for PD patients
Funayama et al., 2007 [13]	448/52 (11.6%) (2 homozygous cases)	457/22 (4.8%)	Japanese	OR for the frequency of A allele 2.63, 95% CI: 1.56–4.35, *p* = 1.24 × 10^−4^	0.06
Di Fonzo at al., 2006 [6]	608/61 (10%)	373/18 (4.8%)	Han Chinese from Taiwan	OR = 2.24, 95% CI: 1.29–3.88, *p* = 0.004	0.05
Fung et al., 2006 [17]	305/27 (9%)	176/1 (0.5%)	Han Chinese from Taiwan	16.99, 95% CI: 2.29 to 126.21, *p*=0.0002	0.4 No positive cases with FH
An et al., 2008 [21]	600/71 (1 homozygous) (11.9%)	334/11 (3.3%)	Han Chinese	OR 3.9, 95% CI = 2.1–7.5, *p* < 0.01	0.06
Tan et al., 2007 [18]	494/37 (7.27%) (1 homozygous)	495/18 (3.64%)	Ethnic Chinese	OR 2.1, 95% CI: 1.1–3.9, *p*=0.014	PAR of 4% for the Gly2385Arg heterozygous genotype.
Tan et al., 2007 [8]	166/2 (Malays)	306/2 (Malays)	Malay 98/173, Indian ethnicity 66/133	OR 2.83, 95% CI 0.40, 20.2, *p*_0.3	0.003 for Malays
Farrer et al., 2007 [16]	410/34	335/13 (3.9%)	Ethnic Chinese	OR 2.24 95% CI 1.16–4.32, *p* < 0.014	MAF 0.08 23.1% (*n* = 6/26) of patients with familial parkinsonism.
Ross et al., 2011 [23]	1,376	962	Japan, Korea, Taiwan	OR: 1.73, 95% CI: 1.20–2.49, *p*=0.0026	MAF 3.3%
	Japan PD: 173	Control: 75			
	Korea |*PD: 844*	*Control: 587*			
	*Taiwan* *PD: 369*	*Control: 300*			
Mata et al., 2005 [20]	100 probands with PD FH/2 cases		Taiwan	1 family with 2 members	
Zabetian et al., 2009 [24]	601/69 (11.5%)	1628/101 (6.2%)	Japanese	OR, 1.83; 95% CI: 1.31–2.54; *p* = 3.3 × 10^−4^	
Gapalai et al., 2015 [14]	695	507	Malaysian	OR 2.22 (*p*=0.019)	MAF = 0.026

OR–odds ratio, MAF–minor allele frequency, PAR–population attributable risk, and FH–family history.

**Table 7:** Studies investigating LRRK2 p.Ala419Val variant in Asian populations.

	Cases	Controls	Ethnicity	OR	MAF for patients/controls
Di Fonzo et al., 2006 [6]	582/10	341/3	Han Chinese from Taiwan	1.95 (0.53–7.15), *p* > 0.05	0.008/0.004
Nuytemans et al., 2009 [27]	620/1	540/0	Belgian	*p* > 0.05	
Jasinska-Myga, 2010 [12]	165/0	364/0	Arab-Berber ethnicity	n/a	
Tan et al., 2010 [28]	250/0	250/0	Han Chinese	n/a	
Ross et al., 2011 [23]	1,376	962	Japan, Korea, Taiwan	OR: 2.27, 95% CI: 1.35–3.83, *p*=0.0011	
	Japan PD: 173	Control: 75		Japan: OR 1.26 (0.38 to 4.22)	
	Korea PD: 844	Control: 587		Korea: OR 2.21 (1.2 to 4.06)	
	Taiwan PD: 369	Control: 300		Taiwan: OR 7.51 (0.95 to 59.6)	
Li et al., 2012 [29]	729/22	585/4	Han Chinese	OR, 4.14; 95% CI: 1.53–12.74	0.015
Wu et al., 2012 [30]	1517/13	1487/13	Han Chinese from China and Singapore Taiwanese	0.98 (0.45 to 2.18)	0.004
Gopalai et al., 2013 [31]	404/1	424/3	Chinese (223/236), Malay (122/110), and Indian (59/78)	0.35, 95% CI: 0.01 to 3.79; *p*=0.624	0.002 cases 0.004 Controls
Heckman et al., 2013 [32]	369/10	300/1	Taiwan, South Korea, Japan	OR 8.33 (1.06–65.43)	Taiwanese 0.013 Japanese – 0.026 South Korea 0.029
Wu-Chou et al., 2013 [33]	626/0	*473/0*	*Han Chinese from Taiwan*	n/a	
Li et al., 2015 [26]	500/18	574/9	*Chinese*	OR 2.57, 95% CI: 1.13–5.86, *p*=0.025	0.018

OR–odds ratio and MAF–minor allele frequency.

## Data Availability

VCF files from exome sequencing used to support the findings of this study are available from the corresponding author upon request.

## Supplementary Materials

Supplementary Materials S1: the 8 LRRK2 SNPs and surrounding 50 base-pairs annotated in Ensembl genome browser S2: calculations of allelic and genotypic frequencies, odds ratios, and Hardy–Weinberg equilibrium S3: chromatogram for Ala419Val homozygous variant with family segregation S4: the results of KASP analysis.
