# Supplementary material for: LRRK2 Mutations and Asian Disease-Associated Variants in the First Parkinson's Disease Cohort from Kazakhstan
Source: Parkinsons Dis. 2020 Feb 19;2020:2763838. doi: 10.1155/2020/2763838 (PMC7049866; doi:10.1155/2020/2763838)
Supplement: Supplementary Materials — S1: the 8 LRRK2 SNPs and surrounding 50 base-pairs annotated in Ensembl genome browser S2: calculations of allelic and genotypic frequencies, odds ratios, and Hardy–Weinberg equilibrium S3: chromatogram for Ala419Val homozygous variant with family segregation S4: the results of KASP analysis. [file 2763838.f1.zip › 2763838.f1/Supplementary 2.docx]

**Supplement 2.** Calculations of allelic and genotypic frequencies, Odds ratios, and Hardy-Weinberg equilibrium

rs35801418

Cases - Plate 1 – 86

Plate 2 – 91

Plate 3 – 56

Total = 239

Uncalled – 7 samples

| GENOTYPES | | |  | Alleles | | |
| --- | --- | --- | --- | --- | --- | --- |
|  | A/A | A/G | G/G | | A | G |
|  |  |  |  | |  |  |
| number | 239 | 0 | 0 | | 478 |  |
| percentage | 1 | 0,0 | 0,0 | | 1 | 0,0 |

Controls- Plate 1 – 92

Plate 2 – 93

Plate 3 – 10

Total = 196

Uncalled – 4 samples

| GENOTYPES | | |  | Alleles | | |
| --- | --- | --- | --- | --- | --- | --- |
|  | A/A | A/G | G/G | | A | G |
|  |  |  |  | |  |  |
| number | 196 | 0 | 0 | | 392 | 0 |
| percentage | 1,0 | 0,00 | 0,0 | | 1,0 | 0,0 |

rs34637584

Cases - Plate 1 – 87

Plate 2 – 93

Plate 3 –56

Total = 241

Uncalled – 5 samples

| GENOTYPES | | |  | Alleles | | |
| --- | --- | --- | --- | --- | --- | --- |
|  | G/G | G/A | A/A | | G | A |
|  |  |  |  | |  |  |
| number | 241 | 0 | 0 | | 482 | 0 |
| percentage | 1,0 | 0,0 | 0,0 | | 1,0 | 0,0 |

Controls- Plate 1 – 93

Plate 2 – 91

Plate 3 – 12

Total = 198

Uncalled – 2 samples

| GENOTYPES | | |  | Alleles | | |
| --- | --- | --- | --- | --- | --- | --- |
|  | G/G | G/A | A/A | | G | A |
|  |  |  |  | |  |  |
| number | 198 | 0 | 0 | | 396 | 0 |
| percentage | 1,0 | 0,00 | 0,0 | | 1,0 | 0,0 |

rs35870237

Cases - Plate 1 – 85

Plate 2 –93

Plate 3 – 55

Total = 242

Uncalled – 4 samples

| GENOTYPES | | |  | Alleles | | |
| --- | --- | --- | --- | --- | --- | --- |
|  | T/T | T/C | C/C | | T | C |
|  |  |  |  | |  |  |
| number | 242 | 0 | 0 | | 484 | 0 |
| percentage | 1,0 | 0,0 | 0,0 | | 1,0 | 0,0 |

Controls - Plate 1 – 91

Plate 2 – 92

Plate 3 – 12

Total = 198

Uncalled – 2 samples

| GENOTYPES | | |  | Alleles | | |
| --- | --- | --- | --- | --- | --- | --- |
|  | T/T | T/C | C/C | | T | C |
|  |  |  |  | |  |  |
| number | 198 | 0 | 0 | | 396 | 0 |
| percentage | 1,0 | 0,0 | 0,0 | | 1,0 | 0,0 |

rs74163686

Cases - Plate 1 – 86

Plate 2 –94

Plate 3 – 56

Total = 240

Uncalled – 6 samples

| GENOTYPES | | |  | Alleles | | |
| --- | --- | --- | --- | --- | --- | --- |
|  | A/A | A/C | C/C | | A | C |
|  |  |  |  | |  |  |
| number | 240 | 0 | 0 | | 480 | 0 |
| percentage | 1,0 | 0,0 | 0,0 | | 1,0 | 0,0 |

Controls - Plate 1 – 93

Plate 2 –93

Plate 3 – 12

Total = 198

Uncalled – 2samples

| GENOTYPES | | |  | Alleles | | |
| --- | --- | --- | --- | --- | --- | --- |
|  | A/A | A/C | C/C | | A | C |
|  |  |  |  | |  |  |
| number | 198 | 0 | 0 | | 396 | 0 |
| percentage | 1,0 | 0,0 | 0,0 | | 1,0 | 0,0 |

rs34995376

Cases - Plate 1 – 77

Plate 2 –94

Plate 3 – 56

Total = 240

Uncalled – 6 samples

| GENOTYPES | | |  | Alleles | | |
| --- | --- | --- | --- | --- | --- | --- |
|  | G/G | G/A | A/A | | G | A |
|  |  |  |  | |  |  |
| number | 240 | 0 | 0 | | 480 | 0 |
| percentage | 1,0 | 0,0 | 0,0 | | 1,0 | 0,0 |

Controls - Plate 1 – 94

Plate 2 –93

Plate 3 – 12

Total = 199

Uncalled – 1 samples

| GENOTYPES | | |  | Alleles | | |
| --- | --- | --- | --- | --- | --- | --- |
|  | G/G | G/A | A/A | | G | A |
|  |  |  |  | |  |  |
| number | 199 | 0 | 0 | | 398 | 0 |
| percentage | 1,0 | 0,0 | 0,0 | | 1,0 | 0,0 |

rs34778348

Cases - Plate 1 – 85

Plate 2 – 94

Plate 3 –54

Total = 239

Uncalled – 7 samples

| GENOTYPES | | |  | Alleles 478 | | |
| --- | --- | --- | --- | --- | --- | --- |
|  | G/G | G/A | A/A | | G | A |
|  |  |  |  | |  |  |
| number | 236 | 3 | 0 | | 475 | 3 |
| percentage | 0,987 | 0,013 | 0,0 | | 0,993 | 0,007 |

Controls - Plate 1 – 92

Plate 2 – 92

Plate 3 –12

Total = 199

Uncalled – 1 samples

| GENOTYPES | | |  | Alleles 398 | | |
| --- | --- | --- | --- | --- | --- | --- |
|  | G/G | G/A | A/A | | G | A |
|  |  |  |  | |  |  |
| number | 197 | 2 | 0 | | 396 | 2 |
| percentage | 0,99 | 0,01 | 0,0 | | 0,995 | 0,005 |

HWE calculation rs34778348

|  | Cases | |  | Controls | |
| --- | --- | --- | --- | --- | --- |
| Genotypes | *Observed # | Expected # |  | *Observed # | Expected # |
| Homozygote reference: | 236 | 236,0 |  | 197 | 197,0 |
| Heterozygote: | 3 | 3,0 |  | 2 | 2,0 |
| Homozygote variant: | 0 | 0,0 |  | 0 | 0,0 |
| Var allele freq: | 0.01 | 239 |  | 0.01 | 199 |
| Chi-squared value = | 0.009533518 |  |  | 0.005076013 |  |
| Chi-squared test P value = | 0.922218 |  |  | 0.943202 |  |

Odds ratio

| Odds ratio | 1.2521 |
| --- | --- |
| 95 % CI: | 0.2071 to 7.5688 |
| z statistic | 0.245 |
| Significance level | P = 0.8065 |

rs34594498

Cases - Plate 1 – 90

Plate 2 –94

Plate 3 –56

Total = 242

Uncalled – 4 samples

| GENOTYPES | | |  | Alleles 484 | |
| --- | --- | --- | --- | --- | --- |
|  | C/C | C/T | T/T | C | T |
|  |  |  |  |  |  |
| number | 233 | 8 | 1 | 474 | 10 |
| percentage | 0,963 | 0,033 | 0,004 | 0,98 | 0,02 |

Controls - Plate 1 – 93

Plate 2 –93

Plate 3 –12

Total =199

Uncalled – 1 samples

| GENOTYPES | | |  | Alleles 398 | | |
| --- | --- | --- | --- | --- | --- | --- |
|  | C/C | C/T | T/T | | C | T |
|  |  |  |  | |  |  |
| number | 194 | 5 | 0 | | 393 | 5 |
| percentage | 0,975 | 0,025 | 0,00 | | 0,988 | 0,012 |

HWE calculation rs34594498

|  | Cases | |  | Controls | |
| --- | --- | --- | --- | --- | --- |
| Genotypes | *Observed # | Expected # |  | *Observed # | Expected # |
| Homozygote reference: | 233 | 232,1 |  | 194 | 194,0 |
| Heterozygote: | 8 | 9,1 |  | 5 | 4,9 |
| Homozygote variant: | 1 | 0,1 |  | 0 | 0,0 |
| Var allele freq: | 0,02 | 242 |  | 0.01 | 199 |
| Chi-squared value = | 8.115179547 |  |  | 0.03221128 |  |
| Chi-squared test P value = | 0.004390 |  |  | 0.857565 |  |

|  | Cases and controls with Homozygous | |  | Controls and controls without homoz | |
| --- | --- | --- | --- | --- | --- |
| Genotypes | *Observed # | Expected # |  | *Observed # | Expected # |
| Homozygote reference: | 427 | 426.1 |  | 427 | 427.1 |
| Heterozygote: | 13 | 14.7 |  | 13 | 12.8 |
| Homozygote variant: | 1 | 0.1 |  | 0 | 0.1 |
| Var allele freq: | 0.02 | 441 |  | 0.01 | 440 |
| Chi-squared value = | 6.175826918 |  |  | 0.09892389 |  |
| Chi-squared test P value = | 0.012951 |  |  | 0.753125 |  |

ODDS ratio calculation

| Odds ratio | 1.4987 |
| --- | --- |
| 95 % CI: | 0.4941 to 4.5463 |
| z statistic | 0.715 |
| Significance level | P = 0.4748 |

rs33949390

Cases - Plate 1 – 83

Plate 2 –94

Plate 3 –56

Total = 236

Uncalled – 8 samples

| GENOTYPES | | |  | Alleles | | |
| --- | --- | --- | --- | --- | --- | --- |
|  | G/C | G/C | C/C | | G | C |
|  |  |  |  | |  |  |
| number | 236 | 0 | 0 | | 472 | 0 |
| percentage | 1,0 | 0,0 | 0,0 | | 1,0 | 0,0 |

Controls - Plate 1 – 94

Plate 2 –93

Plate 3 –11

Total = 199

Uncalled – 1 samples

| GENOTYPES | | |  | Alleles 398 | | |
| --- | --- | --- | --- | --- | --- | --- |
|  | G/C | G/C | C/C | | G | C |
|  |  |  |  | |  |  |
| number | 197 | 2 | 0 | | 396 | 2 |
| percentage | 0,99 | 0,01 | 0,0 | | 0,995 | 0,005 |

HWE calculation rs33949390

|  | Cases | |  | Controls | |
| --- | --- | --- | --- | --- | --- |
| Genotypes | *Observed # | Expected # |  | *Observed # | Expected # |
| Homozygote reference: | 236 |  |  | 199 | 199,0 |
| Heterozygote: | 0 |  |  | 2 | 2,0 |
| Homozygote variant: | 0 |  |  | 0 | 0,0 |
| Var allele freq: |  |  |  | 0.01 | 199 |
| Chi-squared value = |  |  |  | 0.005025 | 201 |
| Chi-squared test P value = |  |  |  | 0.943488 |  |
