# Supplementary material for: LRRK2 Mutations and Asian Disease-Associated Variants in the First Parkinson's Disease Cohort from Kazakhstan
Source: Parkinsons Dis. 2020 Feb 19;2020:2763838. doi: 10.1155/2020/2763838 (PMC7049866; doi:10.1155/2020/2763838)

**Supplement 3**

Chromatogram for Ala419Val homozygous variant with family segregation


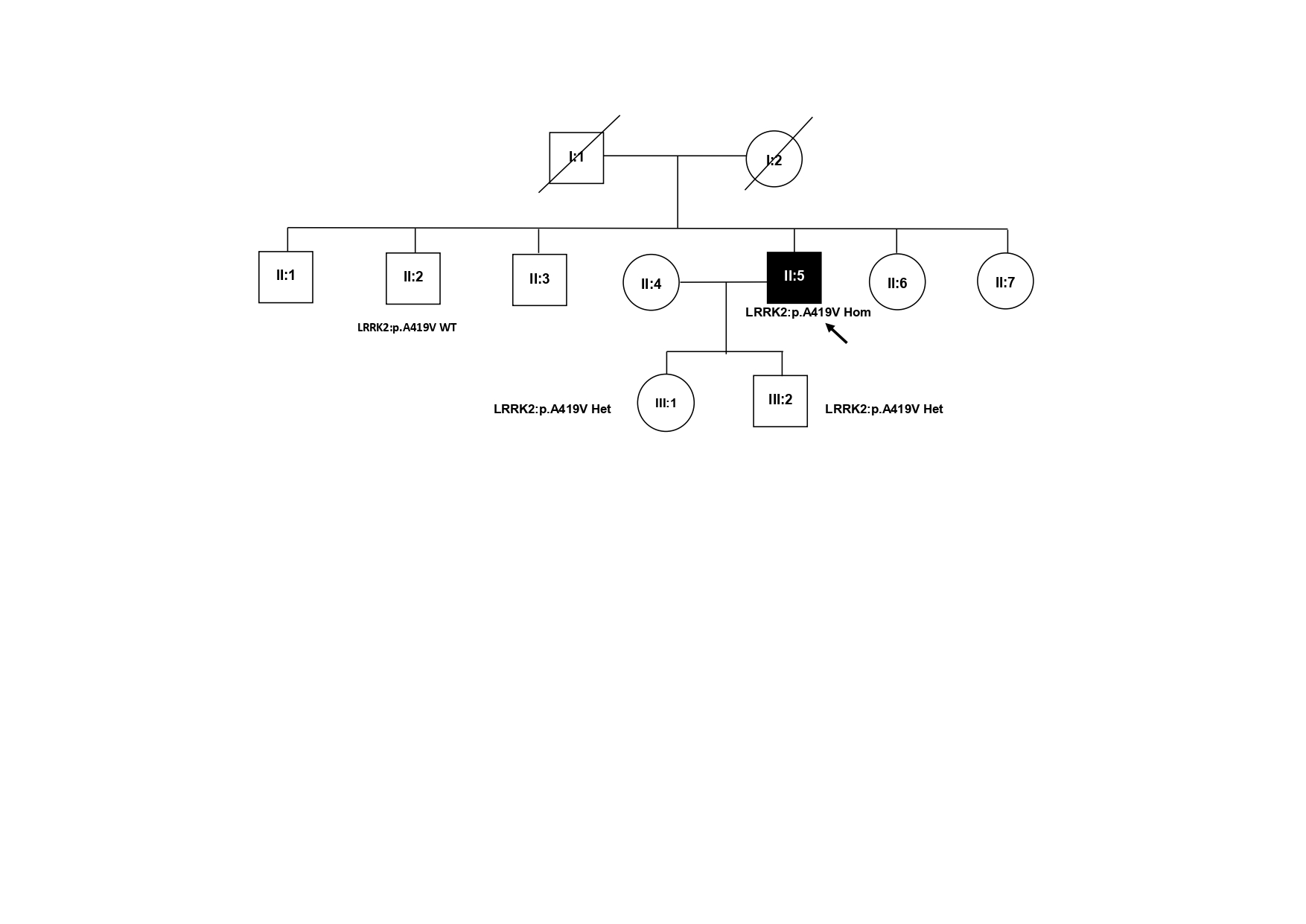


1. II:5 Chromatogram


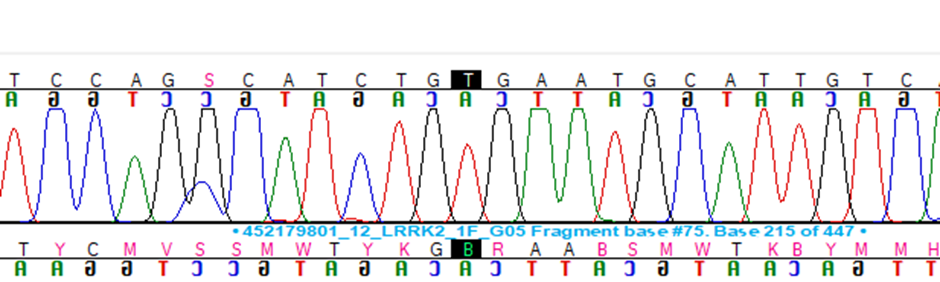


1. III:1 and III:2 Chromatograms


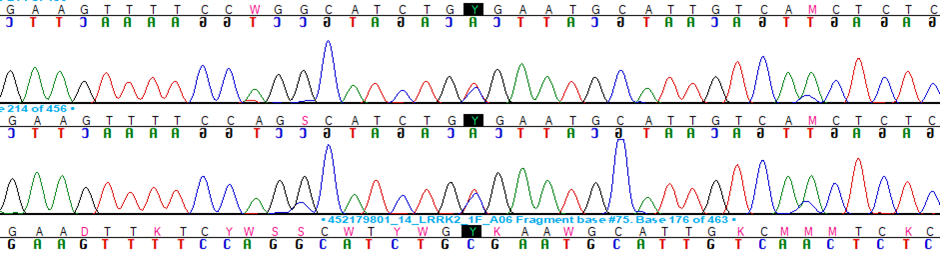


3) II:2 Chromatograms


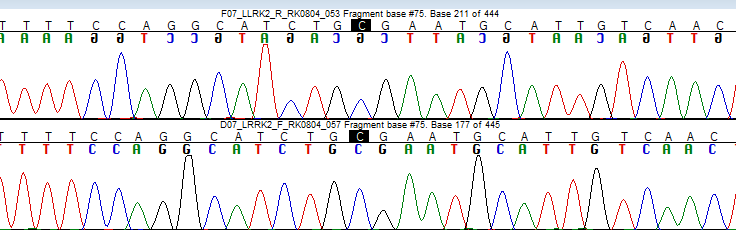

Supplement: Supplementary Materials — S1: the 8 LRRK2 SNPs and surrounding 50 base-pairs annotated in Ensembl genome browser S2: calculations of allelic and genotypic frequencies, odds ratios, and Hardy–Weinberg equilibrium S3: chromatogram for Ala419Val homozygous variant with family segregation S4: the results of KASP analysis. [file 2763838.f1.zip › 2763838.f1/Supplementary 3.docx]
